# Evidence That the EphA2 Receptor Exacerbates Ischemic Brain Injury

**DOI:** 10.1371/journal.pone.0053528

**Published:** 2013-01-07

**Authors:** John Thundyil, Silvia Manzanero, Dale Pavlovski, Tanya R. Cully, Ker-Zhing Lok, Alexander Widiapradja, Prasad Chunduri, Dong-Gyu Jo, Chie Naruse, Masahide Asano, Bradley S. Launikonis, Christopher G. Sobey, Mark G. Coulthard, Thiruma V. Arumugam

**Affiliations:** 1 School of Biomedical Sciences, University of Queensland, St Lucia, Queensland, Australia; 2 School of Pharmacy, Sungkyunkwan University, Suwon, Korea; 3 Division of Transgenic Animal Science, Advanced Science Research Center, Kanazawa University, 13-1 Takara-machi, Kanazawa, Japan; 4 Vascular Biology and Immunopharmacology Group, Department of Pharmacology, Monash University, Clayton, Victoria, Australia; 5 Academic Discipline of Paediatrics and Child Health, University of Queensland, Royal Children’s Hospital, Herston, Queensland, Australia; 6 Paediatric Intensive Care Unit, Royal Children's Hospital, Herston, Queensland, Australia; 7 Queensland Children’s Medical Research Institute, Royal Children's Hospital, Herston, Queensland, Australia; Julius-Maximilians-Universität Würzburg, Germany

## Abstract

Ephrin (Eph) signaling within the central nervous system is known to modulate axon guidance, synaptic plasticity, and to promote long-term potentiation. We investigated the potential involvement of EphA2 receptors in ischemic stroke-induced brain inflammation in a mouse model of focal stroke. Cerebral ischemia was induced in male C57Bl6/J wild-type (WT) and EphA2-deficient (EphA2^−/−^) mice by middle cerebral artery occlusion (MCAO; 60 min), followed by reperfusion (24 or 72 h). Brain infarction was measured using triphenyltetrazolium chloride staining. Neurological deficit scores and brain infarct volumes were significantly less in EphA2^−/−^ mice compared with WT controls. This protection by EphA2 deletion was associated with a comparative decrease in brain edema, blood-brain barrier damage, MMP-9 expression and leukocyte infiltration, and higher expression levels of the tight junction protein, zona occludens-1. Moreover, EphA2^−/−^ brains had significantly lower levels of the pro-apoptotic proteins, cleaved caspase-3 and BAX, and higher levels of the anti-apoptotic protein, Bcl-2 as compared to WT group. We confirmed that isolated WT cortical neurons express the EphA2 receptor and its ligands (ephrin-A1–A3). Furthermore, expression of all four proteins was increased in WT primary cortical neurons following 24 h of glucose deprivation, and in the brains of WT mice following stroke. Glucose deprivation induced less cell death in primary neurons from EphA2^−/−^ compared with WT mice. In conclusion, our data provide the first evidence that the EphA2 receptor directly contributes to blood-brain barrier damage and neuronal death following ischemic stroke.

## Introduction

The Ephrin (Eph) receptor-ligand family is the largest family of tyrosine kinases and it plays a pivotal role during embryogenesis by regulating cell-cell interaction in tissue development [1]–[4]. There are ten Class A Eph receptors (EphA1–EphA10), six Class B Eph receptors (EphB1–EphB6), and nine membrane-anchored ephrin ligands (ephrin-A1–A5 subtypes and ephrin-B1–B3 subtypes) identified in the mammalian genome [5], [6]. The Eph/ephrin receptor-ligand interactions are promiscuous within each within each A or B subclass, with variations in binding affinities, although EphB2 only binds ephrin-B2. There are also exceptions in the binding preferences between A and B class, as EphA4 binds to ephrin-B ligands (ephrin-B2, ephrin-B3) and EphB2 binds to ephrin-A5 [7]–[9]. Within the central nervous system (CNS), the role of Eph/ephrin signaling in development has primarily been studied in relation to axon guidance. It has also been shown that these kinases modulate synaptic plasticity, promote long-term potentiation [10], [11], and can mediate angiogenesis during wound healing and tumor pathogenesis [12]–[15].

The EphA2 receptor has been shown to mediate inflammation during injury, ischemia and other chronic inflammatory conditions [16]–[18]. Recent studies indicate that EphA2 receptor activation contributes to inflammation by promoting vascular permeability in various murine models of injury [19]–[21]. Activation of EphA2 by its highest affinity ligand, ephrin-A1 (recombinant form), led to disassembly of tight junctions in human brain microvascular endothelial cells (HBMEC), whereas EphA2 inactivation prevented this disruption and promoted tight junction formation [22]. These findings are particularly relevant for ischemic stroke, in which damage to cerebral endothelium and disruption of tight junctions leads to altered blood-brain barrier (BBB) permeability, contributing to post-stroke inflammation and edema. In addition, expression of both the EphA2 receptor and ephrin-A1 are known to be upregulated in various models of CNS injury [5], [6], [23]. However, it is unknown whether the EphA2 receptor plays a role in stroke-induced BBB breakdown and injury. Here we demonstrate that deletion of the EphA2 receptor protects against stroke-induced BBB damage, neuronal cell death and infarct development, and adverse neurological outcomes.

## Results

### EphA2^−/−^ Mice Demonstrate Reduced Infarct Volume and Neurological Deficits

**Figure 1:**
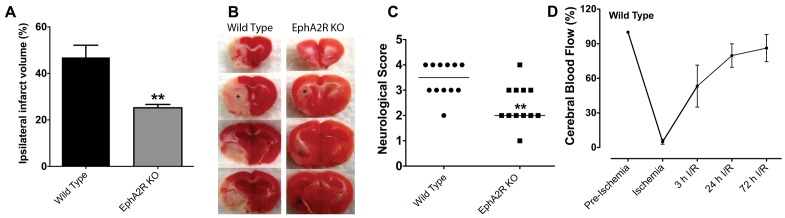
EphA2^−/−^ mice have reduced brain damage and better functional outcome after focal cerebral ischemia. Wild type and EphA2^−/−^ mice were subjected to 60 min MCAO, followed by 72 h reperfusion. (A) Quantified infarct volume was significantly smaller in EphA2^−/−^ compared with WT mice. (B) Representative ipsilateral stroke infarct volumes detected using TTC staining are shown. White (unstained) areas indicate infarction; red (stained) areas indicate normal tissue. (C) A five point neurological score was applied to the wild type and EphA2^−/−^ mice following ischemia and reperfusion. Data are means ± SEM from 10 to 12 animals. **p<0.01 relative to wild type. (D) Regional cerebral blood flow in WT animals was recorded during and after ischemia and expressed as a percentage of the pre-ischemic value.

Cerebral blood flow (CBF) measurements obtained immediately before and after MCAO indicate that blood flow in the ischemic region was reduced to the same extent (∼90%) by MCAO in all animal groups. EphA2 receptors have been documented to promote inflammation in various animal models [19]–[21]. Here, we investigated their potential involvement in ischemic stroke-induced brain inflammation following temporary MCAO for 60 min followed by reperfusion for 72 h. Brain infarction was measured using the triphenyltetrazolium chloride (TTC) staining technique. Infarct volumes in the EphA2^−/−^ mice were significantly smaller compared to wild type (WT) controls (Fig. 1A and 1B). Functional outcomes of this cerebral ischemia-reperfusion (I/R) injury were evaluated in a blinded manner by scoring motor movements. EphA2^−/−^ mice had significantly less neurological deficits compared to WT mice (Fig. 1C).

### EphA2^−/−^ Mice are Protected from Blood-brain Barrier Damage and Immune Cell Infiltration

**Figure 2:**
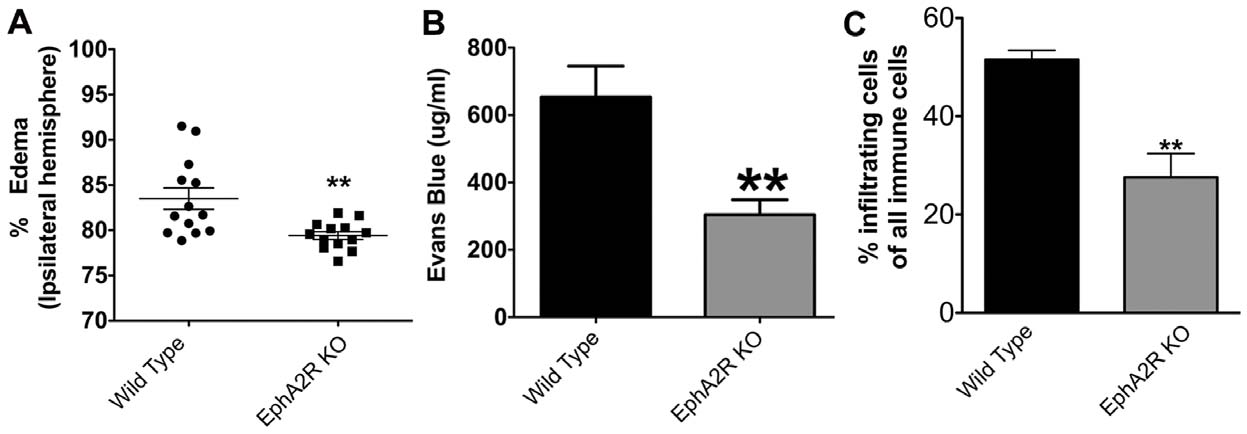
EphA2^−/−^ mice have reduced post-stroke blood-brain barrier (BBB) leakage, edema and infiltrating immune cells. (A) Quantitative measurement of brain edema in ipsilateral brain hemisphere by the wet-dry method. EphA2^−/−^ mice have lower levels of post-stroke brain edema in comparison with wild type mice. (B) Quantitative measurement of Evans Blue dye extravasation in ipsilateral brain hemisphere. Evans Blue dye content was significantly lower in brains from EphA2^−/−^ mice compared with wild type. (C) Flow cytometry analysis of the ipsilateral hemisphere following 72 h of reperfusion showed significantly fewer infiltrating (CD45^high^) immune cells in EphA2^−/−^ mice compared to vehicle treated controls. Data are means ± SEM from 12 to 13 animals. **p<0.01 relative to wild type.

Deletion of the EphA2 receptor results in less permeability across brain endothelial cells *in vitro,* by modulating its tight junction (TJ) protein [22]. Since control of brain endothelial cell permeability is pivotal to BBB integrity, we assessed BBB permeability and leakage using an Evans Blue assay in EphA2^−/−^ and WT mice following temporary MCAO. In comparison to WT, EphA2^−/−^ mice had slightly but significantly less edema in their ipsilateral brain hemispheres (Fig. 2A). Furthermore, EphA2^−/−^ mice also had markedly less Evans Blue leakage than WT controls in the ipsilateral stroke brain tissue exudates, consistent with less BBB damage following cerebral I/R (Fig. 2B). In addition, our data show that cerebral I/R-induced infiltration of CD45^high^ immune cells into the ipsilateral hemisphere after 3 days (Fig. 2C) was significantly attenuated in EphA2^−/−^ mice compared to WT controls.

**Figure 3:**
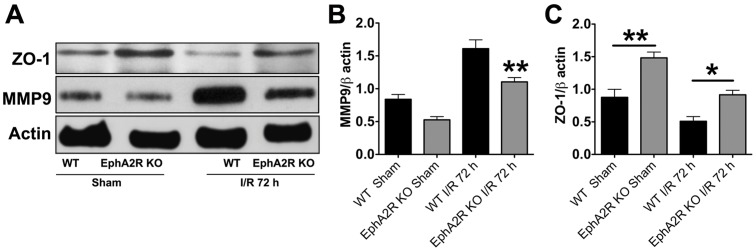
EphA2^−/−^ mice demonstrate less tight junction protein disruption following focal cerebral I/R. Cerebral I/R-induced tight junction disruption and BBB damage were analyzed with zona occludens-1 (ZO-1) and matrix metalloproteinase-9 (MMP-9) antibodies respectively, by immunoblotting. (A and B) EphA2^−/−^ mice demonstrate significantly lower levels of MMP-9 in I/R samples compared to the wild type group, suggestive of lower I/R-induced BBB damage. (A and C) EphA2^−/−^ mice demonstrate significantly higher levels of ZO-1 in sham and I/R samples when compared to the wild type group, indicative of less I/R-induced tight junction disruption. Data are mean ± SEM, n  = 4–6. *p<0.05 or **p<0.01 relative to the wild type controls.

Next we assessed BBB damage by evaluating MMP-9 protein levels in ipsilateral cortical lysates using gel immunoelectrophoresis. Consistent with the Evans Blue assay findings, the immunoblots indicated that the EphA2^−/−^ animals had significantly lower levels of MMP-9 protein compared to WT controls (Fig. 3A and B). Thus, reduced post-stroke BBB damage observed in EphA2^−/−^ mice correlates with their improved functional outcomes. In order to investigate if the greater BBB integrity after I/R in EphA2^−/−^ mice was a direct consequence of EphA2 gene deletion on TJ protein modulation, we assessed expression of the TJ protein, zona occludens-1 (ZO-1). ZO-1 expression was significantly higher in EphA2^−/−^ animals when compared to the WT controls in both sham and post-stroke brain lysates (Fig. 3A and C), thus suggesting that the improved BBB integrity observed in EphA2^−/−^ could perhaps be due to modulation of the ZO-1 tight junction protein, even under non-ischemic conditions.

### Cerebral I/R Injury-induced Pro-apoptotic Cell Death Signaling was Reduced in EphA2^−/−^ Mice

**Figure 4:**
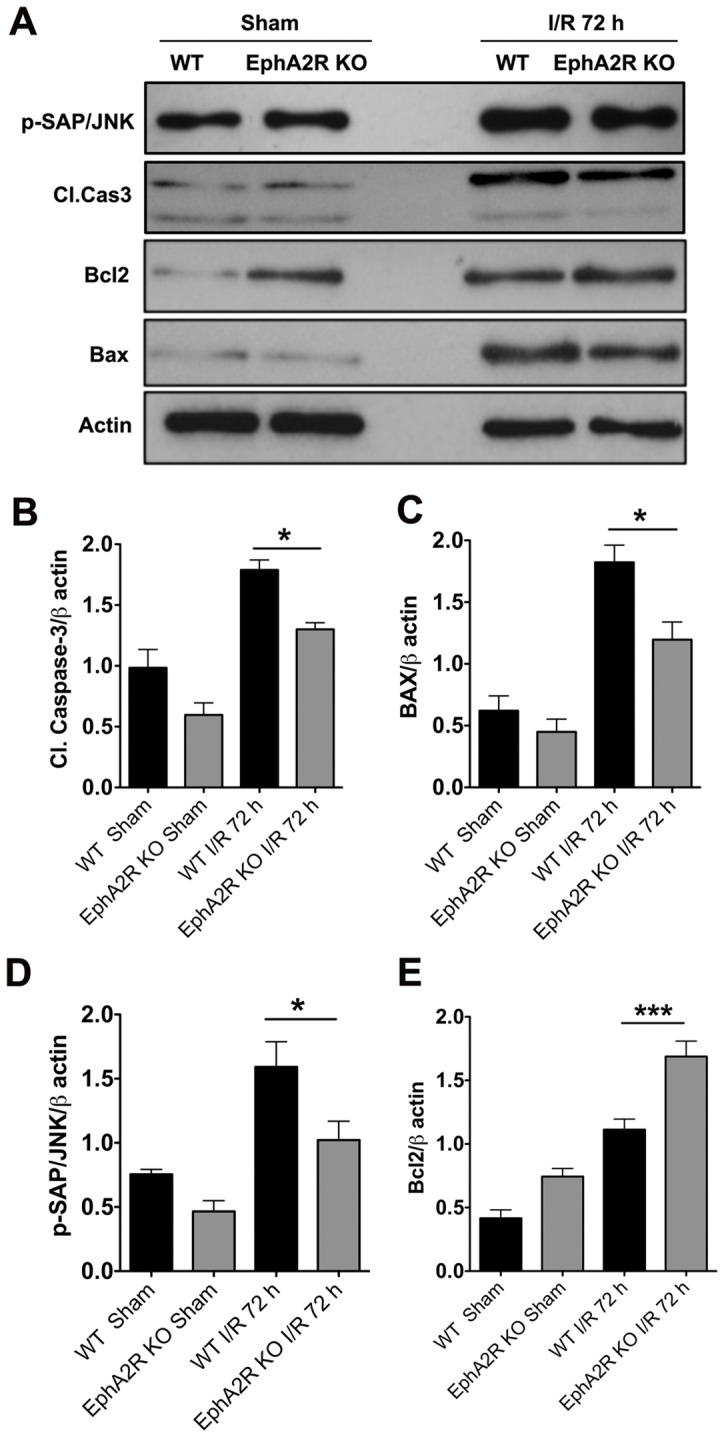
Apoptotic neuronal death is reduced in EphA2^−/−^ mice following focal cerebral I/R. Ipsilateral cortical protein lysates from sham and I/R brains from both wild type and EphA2^−/−^ mice were analyzed for selected pro- and anti-apoptotic proteins using immunoblotting. (A–D) EphA2^−/−^ mice demonstrate significantly lower levels of apoptotic cell death (cleaved caspase-3; Cl. Cas3) and pro-apoptotic proteins (P-SAP/JNK, BAX) as compared to the wild type group following focal cerebral I/R. (A and E) EphA2^−/−^ mice demonstrate significantly higher levels of the anti-apoptotic protein, Bcl–2, compared to wild type following cerebral I/R. Data are mean ± SEM, n = 4–5. *p<0.05, ***p<0.0001 relative to wild type.

In order to investigate the molecular mechanisms underlying the protection of EphA2^−/−^ mice following cerebral I/R, we next evaluated the degree of apoptotic death using immunoblotting. We observed that ipsilateral cortical tissue samples from EphA2^−/−^ mice had significantly lower levels of the apoptotic cell death marker, cleaved caspase-3, compared to the WT controls (Fig. 4A and B). We also found that in comparison with WT, EphA2^−/−^ mice had significantly lower levels of the pro-apoptotic protein, BAX (Fig. 4A and C). We previously demonstrated that c-Jun N-terminal kinase (JNK) activation is implicated in ischemia-induced cellular stress responses and consequent apoptotic cell death [24]. Here, in contrast to the robust increase in JNK activation in WT tissue lysates, we found that EphA2^−/−^ mice had significantly lower levels of P-SAP/JNK levels following cerebral I/R (Fig. 4A and D). Finally, we found that expression of the anti-apoptotic protein, Bcl–2, was significantly higher in EphA2^−/−^ mice compared to WT controls following cerebral I/R (Fig. 4A and E).

### EphA2 Receptors Contribute to Neuronal Cell Death Following Ischemic Conditions *in vitro*

**Figure 5:**
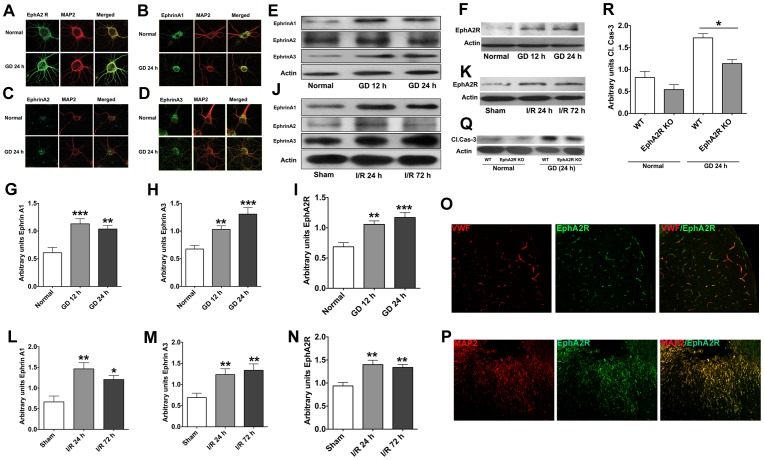
EphA2 expression is modulated following ischemic conditions *in vitro* and *in vivo.* Wild type primary cortical neurons were used to show expression profiles of EphA2 and its ligands - Ephrins A1, 2 and 3 - using immunocytochemistry. (A–D) Cortical neurons showed expression of EphA2 and Ephrins A1–3 under normal and GD conditions. The neuronal protein lysates obtained from normal and GD conditions were probed for changes in the above-mentioned proteins using immunoblotting. (E–I) Expression of EphA2 and its ligands ephrin-A1, ephrin-A2, and ephrin-A3, was increased following GD for 12–24 h when compared to normal conditions. (J–N) A similar increase in expression was observed *in vivo* following cerebral I/R when compared to sham conditions. Cell-specific expression profile of EphA2 in the ischemic (24 I/R) brain sections was analysed in peri-infarct regions (200X) (O–P). EphA2 was primarily localized to neurons and blood vessels in the peri-infarct regions. Protein lysates of cortical neuronal cultures subjected to GD for 24 h were analyzed for the apoptotic cell death marker cleaved caspase-3 (Q–R). EphA2^−/−^ neuronal cultures showed significantly lower levels of cleaved caspase-3 when compared to wild type cultures. Data are mean ± SEM, n = 3–5. *p<0.05, **p<0.001, ***p<0.0001 relative to sham (*in vivo)* and normal controls (*in vitro)* for EphA2, Ephrin A1 or Ephrin A3 blots. Data are mean ± SEM, n = 3. *p<0.05 relative to the wild type controls in Cl.Cas3 blots.

In order to confirm expression of EphA2 and its ligands in cortical neurons, and to evaluate their role in ischemic stroke-induced neuronal death, we first performed confocal imaging. We found that isolated cortical neurons do indeed express the EphA2 receptor and its ligands (ephrin-A1–A3) (Fig. 5A, B, C, D, E and F), and that expressions of ephrin-A1 and ephrin-A3 was greater than that of ephrin-A2 in these cells, consistent with previous reports [5], [6], [23]. Furthermore, expression of all four proteins appeared to be increased in primary cortical neurons following 24 h of glucose deprivation (GD) *in vitro* (Fig. 5E, F, G, H). Of these, the increased levels of EphA2, Ephrin A1 and Ephrin A3 following GD were statistically significant when compared to normal conditions (Fig. 5G, H, I). We next analyzed protein lysates from ipsilateral cortical tissue at 24 or 72 h following cerebral ischemia. Our data showed that expression of the EphA2 receptor and its ligands, ephrin-A1–A3, was indeed upregulated following ischemia when compared to the cortex of a sham-operated animal (Fig. 5J, K, L, M, N). We then explored the cell-specific expression profiles of EphA2 receptor in the WT brain sections (ipsilateral) following I–R (24 h) using immunohistochemistry techniques. We performed immunostaining using specific markers of neurons (Microtubule-associated protein 2; MAP2), astrocytes (Glial fibrillary acidic protein; GFAP), microglia (Ionized calcium binding adaptor molecule 1; Iba1) and endothelial cells (Von Willebrand factor; vWF) observed that EphA2 was predominantly localized in neurons and endothelial cells in the peri-infarct region (Fig. 5O, P). Finally, we tested whether EphA2 receptor deficiency directly affects survival by cortical neurons that are subjected to GD for 24 h in culture. Immunoblot analysis of the protein lysates indicated significantly lower levels of cleaved caspase-3 in neurons from EphA2^−/−^ compared to WT mice, consistent with a direct contributing role of the EphA2 receptor to neuronal cell death following ischemic stroke (Fig. 5Q, R).

## Discussion

This is the first study to identify a role for EphA2 receptors in ischemic stroke pathology. The Eph receptors constitute the largest family of transmembrane tyrosine kinase receptors that bind to their respective membrane-bound ephrin ligands. Within the CNS, the roles of Eph/ephrin receptor-ligand interactions were regarded as being limited to the embryonic development stages, where they mediate growth cone formation and axonal guidance. However, recent studies have shown that these receptors are not mere developmental molecules, and that they can contribute to processes of both health and disease even during adulthood [10]–[15]. EphA2 receptors have been shown to mediate and promote inflammatory processes and angiogenesis during injury, ischemia and other chronic inflammatory conditions, primarily via its actions on endothelial cell permeability [12]–[15].

Cerebral endothelial cells are critical components of the BBB, and their altered permeability following an ischemic stroke plays an important role in promoting secondary brain tissue damage. We hypothesized that deletion of EphA2 receptors would be beneficial to ischemic stroke outcome. Consistent with our hypothesis, we found that the EphA2^−/−^ mice had significantly lower infarct volumes and improved neurological outcomes when compared to age-matched WT mice. In order to explore whether this protective effect was associated with reduced endothelial cell permeability after stroke, we assessed BBB permeability using Evans Blue staining. Consistent with such a mechanism, EphA2^−/−^ mice had significantly lower levels of Evans Blue stain than the WT mice. Similarly the EphA2^−/−^ mice had significantly reduced post-stroke edema and immune cell infiltrates. The importance of immune cell infiltration during stroke has been demonstrated in several species. Leukocytes are involved in the secondary progression of brain damage following stroke and there is typically a dramatic accumulation of neutrophils and macrophages in infarcted tissue during reperfusion [25], an observation recapitulated in our mouse model. Our findings that EphA2^−/−^ mice had significantly reduced inflammatory infiltrate in the brain are consistent with a possible additional mechanism of protection in the EphA2^−/−^ mice.

We further investigated mechanisms related to damage of the BBB cellular matrix after stroke by evaluating the levels of matrix metalloproteinase-9 (MMP-9) [26]. Our findings that MMP-9 levels were reduced in EphA2^−/−^ mice are consistent with their milder post-stroke BBB damage and subsequent leakage, as compared to WT counterparts. Inactivation of EphA2 receptors promotes tight junction formation in brain endothelial cells. We also assessed the expression level of one of the most abundant tight junction proteins, ZO-1. Tight junction proteins are specific to endothelial cells and are responsible for maintaining BBB integrity. We found that EphA2^−/−^ mice had significantly higher levels of ZO-1 protein in both sham-operated and I/R brains compared to WT controls. The higher levels of ZO-1 in the EphA2^−/−^ sham mice suggest that the EphA2^−/−^ mice normally have tighter BBB formations.

Since apoptosis is known to play a major role in penumbral cell death following ischemic stroke [27], we investigated the role of EphA2 receptors on levels of apoptotic proteins and apoptotic cell death in the peri-infarct areas. We found significantly lower levels of pro-apoptotic proteins and apoptotic cell death in EphA2^−/−^ mice compared to WT controls. A similar result was also seen in the activation levels of JNK in EphA2^−/−^ mice compared to WT controls. This reduction in pro-apoptotic signaling could be an outcome of the previously mentioned beneficial effects. Since the overall beneficial effects of reduction in the activation of p-JNK and Cl. Caspase 3 in EphA2^−/−^ mice could not be attributed to specific cell types, we performed immunostaining using specific markers of neurons (MAP2), astrocytes (GFAP), microglia (Iba1) and endothelial cells (VWF). We observed that EphA2 was predominantly localized in neurons and endothelial cells in the peri-infarct region. It is noteworthy that this is one of very few studies to provide a cell-specific expression profile of EphA2 receptors in adult mouse brain. Furthermore, EphA2^−/−^ mice cultures have reduced apoptotic cell death following ischemic conditions in vitro, as compared to WT cultures. This implies a probable direct involvement of EphA2 receptors at the neuronal level in promoting apoptosis-mediated cell death following cerebral ischemia. Although we were unable to fully characterize the cell-specific contributions to this neuroprotection, we have made an earnest effort to address this point. Our findings suggest that the neuroprotective effects seen in the EphA2^−/−^ group could be partially due to a direct effect at the neuronal level and perhaps a greater effect at the endothelial cell level at the BBB. Nevertheless, all these results taken together suggest that deletion of the EphA2 receptor is indeed beneficial within the context of an ischemic stroke.

Complementary findings of upregulated ephrin-A1 and ephrin-A3 ligands and EphA2 receptors in WT brains after stroke, and in primary cortical neurons exposed to GD, probably suggest that activation of EphA2 receptors is important for ephrin-A1/A3 signaling following cerebral ischemia. It has been shown previously that ephrin-A1-mediated activation of the EphA2 receptor can increase cerebral endothelial cell permeability, an acute outcome of cerebral ischemia known to be detrimental for stroke outcome. Similarly, activation of ephrin-A1 and ephrin-A3 in astrocytes has been shown to negatively regulate neuronal regeneration following injury [28]. Thus, while it is plausible that the upregulation of EphA2 receptors and ligands, shown here to occur in response to cerebral ischemia, may ultimately play a beneficial role through promotion of axonal regeneration during longer-term recovery. The present and previous findings are thus consistent with this mechanism playing a clear detrimental role during the early period following cerebral ischemia. In conclusion, this study is the first to investigate the role of EphA2 receptors in ischemic stroke pathology. The results suggest that pharmacological targeting of EphA2 receptors early following cerebral ischemia could be beneficial in limiting tissue damage and neuronal loss.

## Methods

### Animals

The EphA2^−/−^ mice (on C57/BL6J background) used in this study were a kind gift from Dr Yoichuro Iwakura (University of Tokyo, Tokyo, Japan) [29]. The EphA2^−/−^ mice were re-derived at the University of Queensland and back-crossed with C57/BL6J mice for six generations. The C57/BL6J mice were purchased locally from The University of Queensland animal breeding facility. All animals were housed, bred and maintained at the University of Queensland Biological Resources animal facilities, following approval from the Queensland Animal Care and Use Committee. All animal experimental procedures were performed following approval from the University of Queensland Animal Care and Use Committee.

### Primary Mouse Cortical Neuronal Cultures

Cortical neuronal cultures were obtained from neocortical fragments of both WT (C57/BL6) and EphA2 receptor deficient (EphA2^−/−^) mouse embryos using techniques published previously [30]. Briefly, brain cortices were dissected at E16 and incubated in 2 mg/ml trypsin in Ca^2+/^Mg^2+^-free Hank's balanced salt solution (HBSS) (Invitrogen, Carlsbad, CA, USA) buffered with 10 mmol/L HEPES. Following gentle trituration, the tissues were lysed into a homogenous cell suspension that was plated either on poly-L-lysine coated glass cover slips or in 100-mm diameter plastic dishes at a density of 3×l0^4^ cells/cm^2^. They were maintained in neurobasal medium containing B-27 supplements (Invitrogen), 2 mmol/L L-glutamine, 0.001% gentamycin sulfate and 1 mmol/L HEPES (pH 7.2). Cytosine arabinoside (3 µmol/L, Sigma-Aldrich, St. Louis, Missouri, USA) was added to the culture medium to inhibit glial cell proliferation. Cultures were maintained in a humidified incubator at 37°C in a 5% carbon dioxide atmosphere without further media changes for 7 or 8 days. Experiments were performed in 7 to 9-day-old cultures.

### Glucose Deprivation

*In vitro* ischemia-like conditions were achieved by subjecting the neuronal cultures to glucose-deprived (GD) conditions by replacing the normal neurobasal medium with glucose-free Locke’s medium containing (in mmol/L) 154 NaCl, 5.6 KCl, 2.3 CaCl_2_, 1 MgCl_2_ 3.6 NaHCO_3_, 5 HEPES, at pH 7.2, supplemented with gentamycin (5 mg/L; Invitrogen) for 12 or 24 h.

### Immunoblot Analysis

Protein lysates obtained from WT and EphA2^−/−^ mouse cortical neuron cultures, as well as ipsilateral cortical post-stroke brain samples, were subjected to sodium dodecyl sulfate polyacrylamide gel electrophoresis (5–10% SDS-PAGE gels) using a tris-glycine-SDS running buffer. Gels were then electroblotted onto a nitrocellulose membrane (Bio-Rad, Hercules, California, USA), using a semi-dry transfer apparatus (Bio-Rad) containing a mixture of tris-glycine transfer buffer (0.025 mol/L tris base, 0.15 mol/L glycine) and 10% (v/v) methanol for 1.5 h at 15 V. The nitrocellulose membranes were then incubated in blocking buffer (5% non-fat milk in 20 mmol/L tris-HCL, pH 7.5, 137 mmol/L NaCl, 0.2% Tween-20) for 1 h at room temperature and incubated overnight at 4°C with primary antibodies against EphA2, ephrin-A2, A3 and A5 (Santacruz Biotech, Santa Cruz, CA, USA) and A1 (Abcam, Cambridge, UK), cleaved caspase-3, phospho-SAPK/JNK, BAX, Bcl-2 (Cell Signaling, Danvers, Massachusetts, USA), matrix metalloproteinase-9 (MMP-9), zona occludens-1 (ZO-1) (Abcam) and actin (Sigma-Aldrich). After washing three times (5 min per wash) with tris-buffered saline-T (20 mmol/L tris-HCL, pH 7.5, 137 mmol/L NaCl, 0.2% Tween-20), the membrane was incubated with a horseradish peroxidase-conjugated secondary antibody. Following overnight incubation, membranes were washed with tris-buffered saline and incubated with secondary antibodies for 1 h at room temperature. Protein bands were visualized using a chemiluminescence detection kit (Thermo Fischer Scientific Inc., Waltham, MA, USA).

### Immunocytochemistry and Immunohistochemistry

Coverslips containing cortical neurons subjected either to normal neurobasal or Locke’s buffer media (GD conditions) were fixed in 4% buffered PFA in phosphate-buffered saline (PBS; Sigma-Aldrich). Fixed cells were permeabilized and blocked (1% BSA and 0.1% Triton-X in PBS) at room temperature for 1 h before overnight incubation at 4°C with microtubule-associated protein 2 (MAP2, mouse monoclonal, Millipore, Billerica, Massachusetts, USA) along with either EphA2, ephrin-A1, A2, A3 or A5 (rabbit polyclonal, Santacruz Biotech) diluted in blocking solution. Following primary incubation, the coverslips were incubated in the appropriate Alexa Fluor-conjugated secondary antibodies (Invitrogen) for 1 h at room temperature. Following secondary incubation, coverslips were sealed with mounting solution (Sigma-Aldrich) on glass slides. For immunohistochemistry, frozen cryostat brain sections were obtained from stroke mice, following trans-cardiac PFA (4%) perfusion and were stained for the above-mentioned antibodies in a similar fashion. In addition, cell-specific EphA2 expression in PFA-fixed ipsilateral (I/R) brain sections was investigated using cell-specific markers of neurons (MAP2-Microtubule-associated protein 2; mouse monoclonal, Millipore, Billerica, Massachusetts, USA), astrocytes (GFAP-Glial fibrillary acidic protein; mouse monoclonal, Sigma-Aldrich), microglia (Iba1-Ionized calcium binding adaptor molecule 1; goat polyclonal and endothelial cells (VWF-Von Willebrand factor; sheep polyclonal, Abcam).

Imaging was performed using confocal microscopy. Confocal images were taken using an Olympus FV1000 inverted confocal microscope with a 60x water immersion objective (1.0 NA). Single confocal images were recorded onto 512×512 pixel 12bit.tif images. XY images using 488 nm and 633 nm excitation wavelengths with emissions of 500–600 nm and 650–750 nm respectively were acquired simultaneously.

### *In vivo* Stroke Model

A total of 60 WT and 72 of EphA2^−/−^ mice were used in this study. In both WT and EphA2^−/−^ groups, 12–16 week-old (20–24 g) mice were used. Temporary left MCAO was performed for 60 min in both groups in a blinded manner, as previously described [31]. Briefly, the mice were anesthetized using isoflurane and a midline ventral neck incision was made. Unilateral MCAO was performed using a 6–0 nylon monofilament (0.20–0.22 mm tip), followed by reperfusion for 24–72 h. In the sham-operated group, arteries were visualized but not disturbed. CBF measurements were obtained before and after MCAO to determine whether the reduction in flow caused by MCAO differed between the WT and EphA2^−/−^ mice. CBF was measured by using a Doppler probe (Moor LAB, Moor Instruments, UK) placed on the left parietal cortex following a craniotomy. Only mice exhibiting a 90% drop in cerebral blood flow (as this corresponds to dense ischemia) when compared to the baseline were included. Physiological parameters such as temperature (35.7±0.4) were maintained constant for each group during each surgery.

### Neurological Assessment

The functional consequences of focal cerebral I/R injury in each mouse was evaluated using a 5-point neurological deficit scoring scale (0, no deficit; 1, failure to extend right paw; 2, circling to the right; 3, falling to the right; and 4, unable to walk spontaneously). Mice were assessed in a blinded manner and only those mice with a score greater than or equal to 1 following post-surgical awakening were considered stroke-affected and included for analysis. Each mouse was scored every day of experimentation.

### Morphometric Analysis of Infarct Volume

Mice from both groups were euthanized at different time points of reperfusion. Infarct volumes were assessed with 2,3,5-triphenyltetrazolium chloride (TTC, Sigma-Aldrich) stain, using the “indirect” morphometric analysis method, as described previously [32]. Briefly, the brains were sliced into 1-mm coronal sections with the use of a brain matrix (Braintree Scientific, Braintree, MA, USA). The brain sections were incubated in PBS containing 2% TTC at 37°C for 20 min. The infarct volume of each brain slice was determined in a blinded manner using NIH ImageJ software (rsbweb.nih.gov/ij/). The volume of infarct was calculated by summing the infarct area measured in each of the brain slices after correcting for brain swelling, and the infarct percentage was calculated as a percentage of the contralateral structure. Infarct volumes between WT and EphA2^−/−^ groups were compared using analysis of variance, *post hoc* Tukey-Cramer test and Graph Pad Prism (GraphPad Software, La Jolla, CA, USA).

### Measurement of Brain Edema

Degree of stroke-induced brain edema was assessed using the wet-dry method, as described previously [32], [33]. The ipsilateral MCAO cortex was dissected in a humidity chamber and the wet weight was recorded immediately. The ischemic cortex was then incubated at 100°C until a constant dry weight was obtained. Brain edema was analyzed by calculating tissue water content based on the formula: percentage of brain water content = (1-dryweight/wet weight) ×100%.

### Evans Blue Stain Extraction Assay for Blood Brain Barrier Permeability

The BBB permeability was assessed using a modified Evans Blue stain assay in both WT and EphA2^−/−^ stroked mice, following 72 h of reperfusion [34]. Briefly, a 2% Evans Blue solution was prepared in 0.9% saline solution prior to injection. The mice were anesthetized using isoflurane, and the Evans Blue stain solution was injected into the femoral vein (4 ml/kg of body weight dose). Following 30 min circulation time, mice were transcardially perfused with 50 ml of ice-cold PBS (Invitrogen) and brain tissue was dissected and divided into ipsilateral and contralateral hemispheres. Only the ischemic brain hemispheres were assessed for Evans Blue extravasation. The ipsilateral tissue was dried in an oven at 60°C for two days. Dried tissue weight was assessed. The dried ipsilateral tissue was then incubated in formamide solution (volume concentration of 8 ml/g of dry tissue) at 60°C over two days. Following this, formamide containing the extravasated Evans Blue stain was extracted and the stain content in the tissue extract was assessed by reading the absorbance using a spectrophotometer (Thermo Spectronic Genesys 10 UV, Thermo Fischer Scientific Inc.) at 620 nm and quantified according to a standard curve. The Evans Blue concentrations are presented as ug/ml of dried brain tissue extract.

### Flow Cytometry

We performed flow cytometry cell-type analysis on ipsilateral stroked brain cortices obtained from EphA2^−/−^ and WT mice. Mice from both groups having similar neurological scores (>2 or circling) and visible cortical infarcts in the ipsilateral brain hemispheres following 60 min MCAO and 3 days of reperfusion were included. Flow cytometric analyses were performed as previously described [25]. Briefly, three hemispheres from each group were pooled for comparison in each experiment. Cells were stained with Ly6G, CD11b (BD Biosciences, San Jose, CA, USA), CD11c and CD45 (eBioscience, San Diego, CA, USA) to detect surface staining.

### Data Analysis

All results are reported as means ± SEM. The overall significance of the data was examined by one-way analysis of variance (ANOVA). Differences between the groups were considered significant at p<0.05 with the appropriate Bonferroni correction made for multiple comparisons. Neurological behavior scores were analyzed using a nonparametric Kruskal-Wallis test and Dunn’s Multiple Comparison Test.
